# Controls on Dissolved Organic Carbon Bioreactivity in River Systems

**DOI:** 10.1038/s41598-019-50552-y

**Published:** 2019-10-17

**Authors:** Ana R. A. Soares, Jean-François Lapierre, Balathandayuthabani P. Selvam, Göran Lindström, Martin Berggren

**Affiliations:** 1 Department of Physical Geography and Ecosystem Science, Lund University, SE-223 62 Lund, Sweden; 2 University of Montréal, Department of Biological Sciences, Montreal, PQ H3C 3J7 Canada; 3 Department of Thematic Studies, Tema Environmental Change, Linköping University, Linköping, Sweden; 4 Swedish Meteorological and Hydrological Institute, Norrköping, SE-601 76 Sweden

**Keywords:** Carbon cycle, Limnology

## Abstract

Inland waters transport, transform and retain significant amounts of dissolved organic carbon (DOC) that may be biologically reactive (bioreactive) and thus potentially degraded into atmospheric CO_2_. Despite its global importance, relatively little is known about environmental controls on bioreactivity of DOC as it moves through river systems with varying water residence time (WRT). Here we determined the influence of WRT and landscape properties on DOC bioreactivity in 15 Swedish catchments spanning a large geographical and environmental gradient. We found that the short-term bioreactive pools (0–6 d of decay experiments) were linked to high aquatic primary productivity that, in turn, was stimulated by phosphorus loading from forested, agricultural and urban areas. Unexpectedly, the percentage of long-term bioreactive DOC (determined in 1-year experiments) increased with WRT, possibly due to photo-transformation of recalcitrant DOC from terrestrial sources into long-term bioreactive DOC with relatively lower aromaticity. Thus, despite overall decreases in DOC during water transit through the inland water continuum, DOC becomes relatively more bioreactive on a long time-scale. This increase in DOC bioreactivity with increasing WRT along the freshwater continuum has previously been overlooked. Further studies are needed to explain the processes and mechanisms behind this pattern on a molecular level.

## Introduction

Inland waters receive roughly 5.1 Pg of terrestrial carbon (C) per year, which equals to approximately 70% of the global annual terrestrial net ecosystem production^1^. This riverine C flux is mainly land-derived and partly represented by dissolved organic C (DOC)^2^. Several studies have shown that much of the riverine DOC is lost during passage through inland waters^3–5^, but much less is known regarding how riverine DOC bioreactivity (i.e. biological degradability by bacteria) changes as water progresses towards the end of the freshwater continuum. In spite of advances made to integrate the riverine C flux in global C cycling models, and the many efforts to unravel its magnitude, a major knowledge gap remains with regard to the mechanisms that control changes in the bioreactivity of riverine DOC along the freshwater continuum prior to its delivery to coastal waters^6,7^.

Improved understanding of the controls on DOC bioreactivity in continental watersheds is hampered by the lack of studies that simultaneously address water residence time (WRT) and other environmental control factors such as land use and land cover^8^. It is often assumed that WRT in freshwater systems causes a unidirectional decrease in DOC bioreactivity^5,9^, but if internal loadings of bioreactive DOC compensates or even surpasses the biological consumption of DOC in the aquatic network^10^, then downstream coastal ecosystems would receive more bioreactive DOC than expected from current knowledge. This would have major consequences for the function of recipient coastal ecosystems, such as for CO_2_ emissions of coastal waters to the atmosphere, and on coastal hypoxia^6,11^. However, whether or not riverine DOC becomes more bioreactive when it reaches the sea is a fundamental question that is largely unresolved^12^.

Recent studies challenge the view that DOC bioreactivity monotonically decrease across the aquatic continuum. For example, photochemical degradation and other processes may contribute to the replenishment of bioreactive DOC during the time of water transit through continental watersheds^10^. Along the same line, Selvam *et al*.^13^ (conditionally accepted) showed unexpected increases in the DOC photo-degradation potential towards the end of the freshwater continuum at river mouths, which may cause the release of highly bioreactive low molecular weight DOC compounds^14^ such as organic acids, free amino acids and simple carbohydrates. Moreover, it has been shown that lakes with long water residence times may act as sources rather than sinks of DOC in response to release from primary production (PP)^10^, and this newly produced DOC may be highly bioreactive. In rivers, high concentrations of total nitrogen (TN) and total phosphorus (TP) have been found close to river mouths, and were ascribed to nutrient leaching from lowland agricultural and urban land areas^15^. Since primary production in river waters is mostly P-limited^16^, it is possible that high TP concentrations driven by human activity sustain a highly bioreactive DOC pool at the end of the freshwater continuum as a result of DOC derived from P-enhanced PP. Taken together, recent evidence suggests that continental watersheds may sustain DOC bioreactivity along the aquatic continuum, but this hypothesis remains to be tested.

Here, we assessed the influence of both landscape and WRT on DOC bioreactivity at 15 river mouths with catchments spanning a large environmental gradient across Sweden. Based on standardized degradation experiments performed with ambient microbial communities at 20 °C and in dark conditions, we predicted that short-term bioreactive DOC (STBR; degradable within six days) at the end of the freshwater continuum does not decrease with WRT, but rather increases with increases in aquatic PP. Nonetheless, we expected that absolute amounts of long-term bioreactive DOC (LTBR; degradable up to one year) decrease as the bulk pool of terrestrially-derived DOC is degraded with increasing cumulative WRTs.

## Results

**Figure 1:**
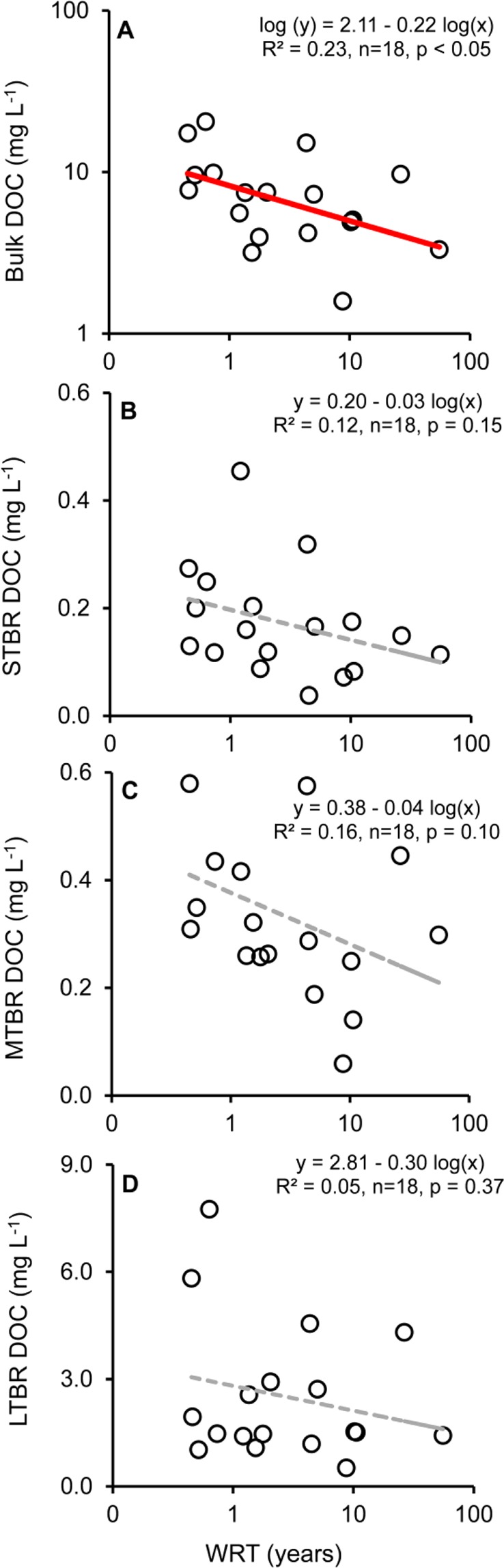
(**a**) Bulk dissolved organic carbon (DOC), (**b**) short-term bioreactive (STBR) DOC, (**c**) medium-term bioreactive DOC (MTBR) and (**d**) long-term bioreactive (LTBR) DOC plotted against water residence time (WRT) on logarithmic scales. Red solid and dashed grey lines, respectively, indicate significant and non-significant relationships.

The bioreactive DOC pools, determined in our dark standard bottle incubations, varied between 0.04–0.45 mg L^−1^ (0.17 ± 0.10; mean ± SD) for the short-term biologically reactive (STBR; 0–6 d of decay experiments), 0.06–0.60 mg L^−1^ (0.34 ± 0.15) for the mid-term biologically reactive (MTBR; 7–22 d of decay experiments) and 0.52–7.75 mg L^−1^ (2.51 ± 1.92) for the long-term biologically reactive (LTBR; 23–365 d of decay experiment). The absolute concentrations of all the bioreactive pools were independent of WRT, whereas bulk DOC concentrations decreased significantly with increased WRT (Fig. 1). We measured other variables such as primary production, coloured dissolved organic matter (CDOM, m^−1^, Naperian units, an index of the concentration of terrestrial coloured DOC measured as the absorption coefficient at 440 nm) and specific ultraviolet absorbance at 254 nm (SUVA_254_, m^−1^, an index of aromaticity of the DOC; Supplementary Table 1). The PP ranged from 2.7 to 40.70 µg C L^−1^ d^−1^ (mean of 18.90 ± 10.60 SD), CDOM from 0.0 to 8.5 m^−1^ (mean of 3.3 ± 2.4) and SUVA_254_ from 0.75 to 5.18 mg C^−1^ m^−1^ (mean of 2.95 ± 1.13). While PP was independent of WRT, SUVA_254_ and CDOM were negatively correlated to WRT (Supplementary Fig. 1). We also determined surface water DOC, TN and TP concentrations which varied greatly among the 18 sampled sites (Table 1).

**Table 1:** Physical, chemical, and land use and land cover statistics for the 18 sampled river sites and their catchments.

Number	System Name	Outlet type	Sampling date	Catchment area (km^2^)	Agriculture (%)	Mountain (%)	Wetland (%)	Lake (%)	Forest (%)	Urban (%)	Other (%)	WRT (years)	TN (mg L^−1^)	TP (µg L^−1^)	DOC (mg L^−1^)
1	Torneträsk	Lake	15/07/2013	3349	0	36	3	14	18	0	30	8.7	0.05	9	0.8
2	Torne älv	River	28/07/2013	39789	0	6	16	5	53	0	19	1.2	0.17	15	5.6
3	Töre älv	River	28/07/2013	449	7	0	1	4	86	2	0	0.5	0.60	20	9.6
4	Alterälven	River	28/07/2013	459	3	0	3	4	89	0	0	0.7	0.52	23	9.9
5	Pite älv	River	28/07/2013	11245	0	0	0	25	62	12	0	1.5	0.08	21	3.2
6	Skellefte älv	River	28/07/2013	11725	1	11	9	13	61	0	5	4.5	0.14	13	4.2
7	Ume älv	River	30/07/2013	26759	1	10	8	8	63	0	10	1.8	0.09	18	4.0
8	Öre älv	River	29/07/2013	3001	2	0	12	3	84	0	0	0.5	0.36	17	7.7
9	Delångersån	River	15/08/2013	1828	4	0	1	12	83	0	0	5.0	0.19	17	7.3
10	Ljusnan	River	15/08/2013	19820	2	1	8	5	77	0	6	1.4	0.28	18	7.5
11	Dalälven	River	15/08/2013	28909	3	0	8	7	77	1	4	2.1	0.07	17	7.5
12	Nyköpingsån	River	03/07/2013	3631	19	0	1	13	66	2	0	4.3	0.67	26	15.2
13	Motala Ström	River	03/07/2013	15393	19	0	1	20	58	2	0	26.5	0.43	24	9.7
14	Vättern	Lake	03/07/2013	6547	15	0	1	35	46	2	0	55.5	0.35	10	3.3
15	Götä älv, Trollhättan	Lake	16/08/2013	47021	11	0	4	19	61	1	4	10.6	0.38	16	4.9
16	Götä älv, Alelyckan	River	16/08/2013	48146	11	0	4	18	62	1	3	10.2	0.45	17	5.1
17	Lyckebeån	River	25/06/2013	802	7	0	1	4	86	2	0	0.6	1.14	29	20.6
18	Helge å	River	25/06/2013	131	19	0	3	5	71	3	0	0.5	1.06	24	16.6

Water residence time, WRT; total nitrogen, TN; total phosphorus, TP; dissolved organic carbon, DOC.

Landscape properties in catchments of the sampled sites were reduced into two main axes of variation, based on a principal component analysis (PCA) that extracted two significant principal components (PC) explaining 73% of the total variance (Supplementary Fig. 2). The PC1 (39.7%) was characterized by negative loadings for forest, urban and agricultural areas, while positive scores were associated with lake, wetland, mountain, and other types of land cover. In turn, PC2 (33.4%) was characterized by positive loadings for land use and cover such as agriculture, urban and lake which mostly resembled the southern catchments (Table 1), while forest, wetland, mountain and other land use types showed negative loadings on PC2. Hereafter, we refer to the patterns in bioreactivity in response to these two components, instead of using the many original land cover variables separate.

**Figure 2:**
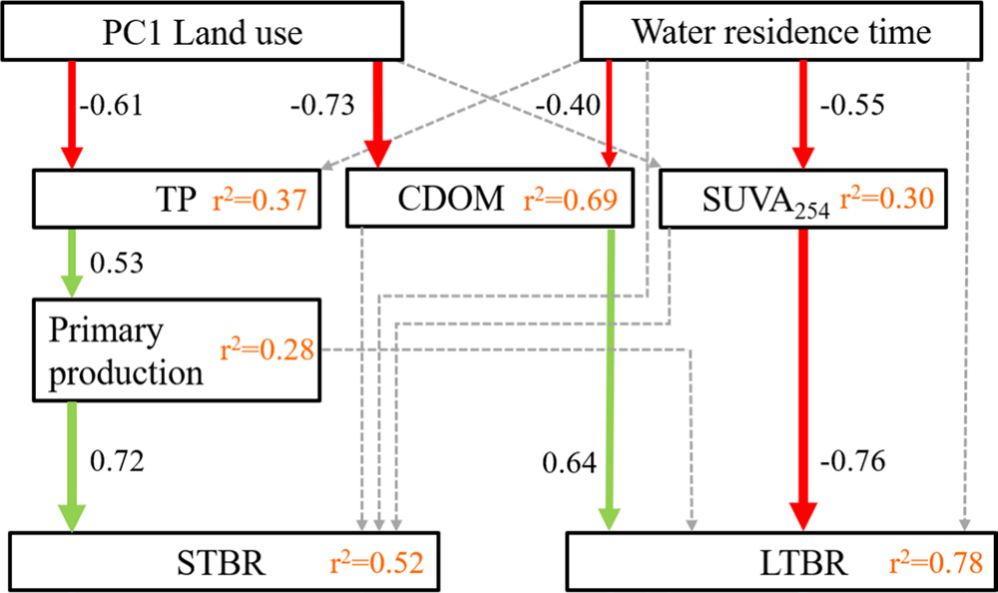
Structural equation model linking short- (STBR) and long-term bioreactive (LTBR) pools (given as absolute concentrations) to land use and water residence time (WRT; given in years). Red arrows show directional pathways negatively related while green arrows show pathways positively related. The coefficients shown alongside the arrows represent the rate at which the response variable changes in response to a change in its predictor. Pathway significance was determined at the *p* < 0.05 level. Dashed light grey arrows denote non-significant pathways (N.S.) which were not included in the final model. χ^2^ = 27.86; P = 0.11; df = 20.

We further determined the effect of land use and WRT on STBR, MTBR and LTBR across our systems using structural equation modeling (SEM; Supplementary Fig. 3). The MTBR pool was excluded from the final model, as we obtained the strongest significant model only with STBR and LTBR (Fig. 2). We found that concentrations of STBR were strongly linked to rates of primary production (r^2^ = 0.52; *p* < 0.05), which were in turn a function of concentrations of TP, and ultimately, of land use and land cover (forest, agriculture and urban). There was no significant direct link between land use and STBR, suggesting an indirect effect of land use and cover on STBR concentrations that is conveyed through loadings of phosphorus and its effect on primary production, which sustained this bioreactive pool at the end of the aquatic continuum. Concentrations of LTBR were explained by both SUVA_254_ and CDOM (r^2^ = 0.78; *p* < 0.05), as LTBR was negatively related to SUVA_254_, but positively related to CDOM. In turn, SUVA_254_ and CDOM were negatively affected by WRT.

**Figure 3:**
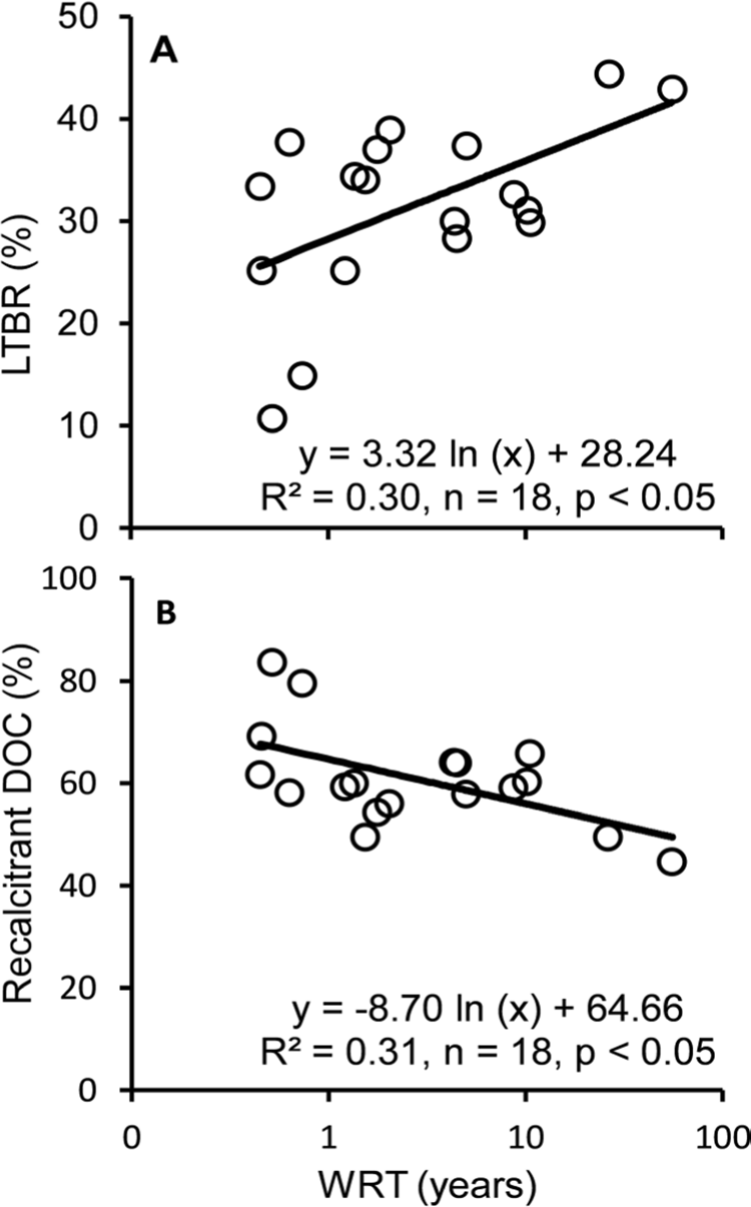
(**a**) Percentage of long-term bioreactivity (LTBR) plotted against the logarithm of water residence time (WRT), and (**b**) percentage of recalcitrant DOC plotted against the logarithm of WRT.

The proportion of LTBR of the total pool (% of DOC) varied between 11% and 44% and increased with longer WRT (Fig. 3a). In contrast, the relative amount of recalcitrant DOC estimated as the difference between the total DOC and the reactive fractions was 45–84% and decreased with longer WRT (Fig. 3b). This shows that the net consequence of the loadings and processing of DOC along the continuum did not lead to a preferential loss of labile DOC, but rather to the replenishment of a bioreactive pool at a rate that surpassed the relative losses.

## Discussion

Our results suggest a substantial replenishment of a biologically reactive DOC pool as water transits from land to sea, possibly as function of photochemical processing and primary production, and ultimately controlled by water residence time (WRT) together with land cover and land use patterns through the aquatic continuum. We found that the share (% of total DOC) of short-term bioreactive DOC at the river mouths was independent of WRT, while the relative long-term bioreactivity (% of DOC) was positively affected by WRT. Our findings improve the understanding of the mechanistic underpinnings of DOC bioreactivity in continental watersheds, and reveal that locally produced bioreactive DOC pool can sustain bacterial activity at the end of the river continuum on both short and long timescales.

The positive effect of aquatic primary production on the absolute concentration of short-term biologically reactive (STBR) DOC observed here agree with previous studies^17–19^, but our results further link this effect to natural land cover and human land use properties along the aquatic continuum. Moreover, we show a dissociation of the STBR DOC from the concentrations of bulk DOC along the WRT gradient, which is in line with the findings from the Hudson river by del Giorgio and Pace^17^ that STBR was primarily influenced by phytoplankton growth while only the DOC bioreactivity at greater timescales was influenced by the bulk terrestrially-originated DOC. Given that bulk DOC concentrations tended to decrease with increasing WRT, our results suggest a relatively higher importance of aquatic primary production for the properties of DOC with increasing transit time in the aquatic continuum. Such a view is consistent with a recent study involving more than 100 000 measurements from rivers across the United States, where a shift from upstream dominance of aromatic terrestrial-like organic matter to downstream gains of aliphatic autochthonous DOC was observed^18^. Similarly, in spite of decreases in bulk DOC the absolute abundance of fluorescent compounds resembling autochthonous DOC was found to be independent of WRT in more than 500 Swedish lakes^19^. In line with the independence of STBR from WRT and given the autochthonous production of STBR linked to land cover and land use change along the continuum, our results compellingly demonstrate that substantial amounts and proportions of the DOC pool that reach Swedish river mouths are biologically reactive at short time scales.

Land use and land cover such as forest, agriculture and urban areas were characteristic of high surface water TP concentrations. While forested catchments have recently been shown to be large contributors of bioavailable phosphorus to inland waters^20^, agriculture and urban areas are well-known sources of nutrients to recipient aquatic systems^21,22^. However, urban areas may alone play an important role for river mouth biogeochemistry since they are often located near river mouths^23^. Subsequently, anthropogenic stimulation of the production of STBR towards the end of the aquatic continuum and right before the coastal environment may contribute to explaining the lack of a decreasing trend in STBR at river mouths (Fig. 1).

The decrease in CDOM but increase in SUVA_254_ and in long-term DOC bioreactivity (% LTBR) with increasing WRT suggest that coloured refractory DOC of terrestrial origin is photo-transformed into long-term reactive DOC during river transit (Fig. 2). Thus, while our findings agree with the notion that terrestrial DOC is primarily made of complex aromatic structures which bacteria have difficulties to degrade^24^, they further demonstrate that during transit through continental watersheds, terrestrial DOC is transformed into less aromatic and more bioreactive forms^25–27^. Catchments with long WRT, where photo-processing can occur to greater extents, thus showed a higher capacity to sustain long-term bacterial activity downstream. Although long WRT could also favor complete oxidation of DOC hence removal of a source of potential LTBR from inland waters, our results show that the role of WRT as facilitator of organic matter transformation was greater than the role of WRT as a factor leading to CDOM removal (Fig. 2).

Our study confirms previous knowledge on an absolute DOC loss and a preferential loss of colored DOC with increasing WRT^4,28^. However, our results further reveal that the relative LTBR concentrations (%) becomes greater over time, which suggests that it increases as DOC transits towards river mouths. Altogether our findings highlight that terrestrial DOC can support long-term bioreactivity in response to processing along rivers networks, becoming more bioavailable with increasing WRT. However, in addition to water residence time, DOC chemical composition and molecular size are known important factors governing reactivity and degradation of DOC in aquatic continuum^29,30^. Further studies are needed to elucidate relationships and interactions between DOM molecular features and the bioavailable fractions that we studied here.

Our study shows a bioreactive organic C pool at river mouths and thus suggests that river mouths are reactive segments of the inland water continuum. From an ecological perspective, the STBR pool may be more relevant than the LTBR pool, as the former pool comprises energy that can at any given moment support bacterial processes. However, from a greenhouse gas emission perspective, the net effect of STBR on gas budgets may be null, as most CO_2_ fixed by primary producers is cycled back to the atmosphere after mineralization. Our results, nonetheless, do not indicate that C derived from primary production was the only source of STBR, but rather that river mouths with higher primary production rates showed higher STBR pools. The LTBR may be biogeochemically more relevant at broad spatial and temporal scales for carbon budgets as it comprises a carbon pool that has been previously fixed in forested soils and that can be potentially mineralized and return to the atmosphere (Cole *et al*. 2007). An overall improved mechanistic understanding of different bioreactive DOC pools can help global numerical models to better constrain current C budgets, and to more accurately forecast how expected changes in land use and WRT patterns can impact the C cycle.

During the past several decades, oxygen concentrations of coastal waters have been decreasing worldwide^31^. Despite availability of monitoring data on oxygen consuming organic matter for some coastal zones, these data are still not sufficient for a complete understanding of oxygen budgets on a global scale^32^. Riverine delivery of organic matter to the coastal zone is one of the main factors driving the current increase of low-oxygen coastal areas globally, which are in turn increasing in number and extent^11^. The deoxygenation of coastal zones is a major threat to biodiversity, productivity, element biogeochemical cycling and for the many ecosystem services provided by coasts^33^. This study contributes to a better understanding of the drivers of the riverine export of oxygen-consuming organic matter across different systems, and it is thus ground for an improved assessment of the role of riverine organic matter loadings on coastal oxygen budgets.

## Conclusion

In summary, our study shows a broad scale pattern of short-term bioreactive DOC replenishment along the continuum through aquatic primary production, while long-term DOC bioreactivity was sustained through reprocessing of terrestrial DOC derived from upstream ecosystems. Our study further made clear that in comparison to WRT, land use is a stronger driver of the potential DOC bioreactivity measured at the end of the freshwater continuum, influencing both the short- and the long-term pools. Our findings show that terrestrial DOC reprocessing maintains a bioreactive DOC pool and that human activity on land use supplies an additional layer of bioreactive DOC that can be exported to coastal waters. Our findings suggest that a higher than expected fraction of the river delivered DOC may be mineralized in the adjacent recipient coastal waters and highlight a causal link between land use in the continental shelves and short- and long-term biological, chemical and physical processes in downstream marine environments.

## Materials and Methods

**Figure 4:**
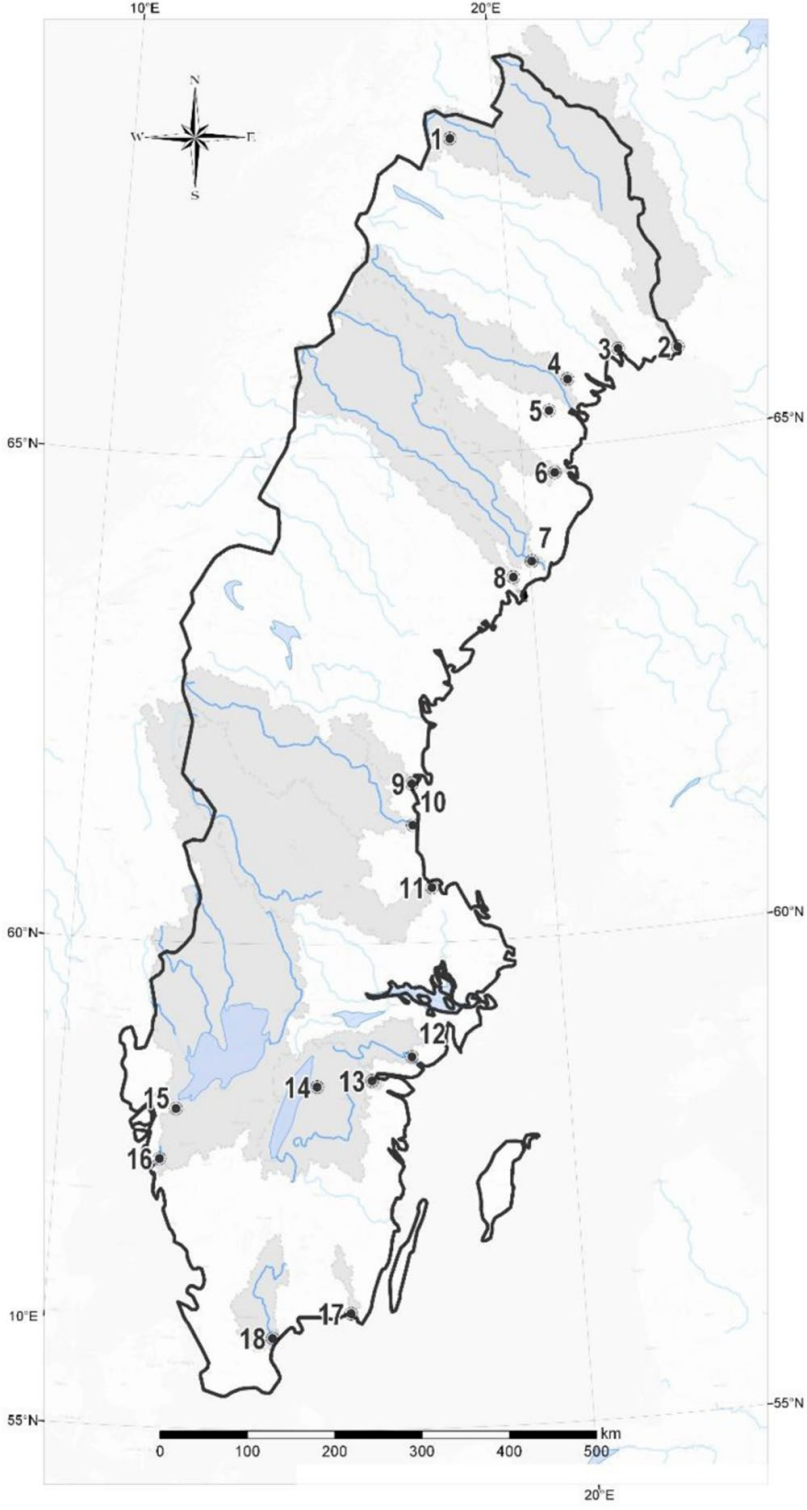
Geographic location of the river outlets sampled. River legend is displayed on Table 1.

We sampled fifteen Swedish river outlets draining either to the Baltic Sea or to the Kattegat once during summer baseflow conditions, between June and August 2013 (Table 1; Fig. 4). In addition, we sampled the upper part of three of the rivers, near the outlets of three large lakes (Vänern, Vättern, Torneträsk. The 18 sampled sites are located between latitudes 56° and 68°N, encompassing a 1800 km north–south climate gradient with a subarctic climate in the north and a temperate climate in the far south (Fig. 4). Catchment size varied between 131 and 48136 km^2^. Proportions of land cover and land use for the area upstream the sampling points were retrieved from the Swedish Meteorological and Hydrological Institute. We included classes such as lake, forest, wetland, urban and agricultural areas and a class which included all remaining land cover and land use types. Typically, landscape varies from more mountainous, wetland and forest-rich areas in the north, to catchments with significant agricultural and urban areas in the south of Sweden (Table 1). We calculated catchment WRT as the average age of the water at each measurement point. We define age as the flow weighted time that water molecules have spent in all upstream rivers and lakes. This is calculated based on estimates of the turnover times in each river and lake, the discharge that flows out of each catchment, and the hydrological network that connects all catchments. The information is based on the S-HYPE model, a hydrological model that describes Sweden in high spatial detail. An earlier version of S-HYPE was described in Strömqvist, *et al*.^34^. The method for calculation of WRT is described in Lindström, *et al*.^35^.

We sampled each system either in the middle of the river or at seven meters from the margin using a peristaltic pump and a 32-mm hose. We attached a grapnel to the hose which was then thrown into the water and fixed to the streambed. The hose inlet floated approximately 30 cm under the water surface. Water for analytical purposes was collected by pumping approximately 40 L of water into two 20-L Thermo Scientific low-density polyethylene cubitainers. Prior to sample collection the cubitainers were acid washed and rinsed three times with sample water. In parallel, we used a 0.45 µm filter (30 mm PES filters, Thermo scientific, MA, USA) and filtered water samples into 40-mL falcon or EPA borosilicate amber glass tubes for analyses of total dissolved fractions of phosphorus, nitrogen and organic carbon. Analysis of phosphorus and nitrogen were carried out at the Evolutionary Biology Center, Uppsala University, following standard methods. We also collected samples for analysis of δ^18^O of dissolved oxygen by filtering water through a 0.2 µm-filter into 12-mL exetainers, which were totally filled and had no oxygen bubbles. Standard water parameters were measured using a YSI 556 multi-parameter chemistry meter (YSI Inc., Yellow Springs, OH, USA). All samples were stored in cooling boxes with ice packs during transport to Lund, which occurred no later than a day after sampling.

After arrival at the laboratory, we immediately initiated DOC bioreactivity incubations. We carried out dark incubations, at 20 °C during 23 days on 1.0 µm-filtered samples (PALL A/E) in duplicate 500-mL Erlenmeyers with ground glass joints. Bioreactivity was estimated from the slope of dissolved oxygen change in the bottles over time, which was measured continuously with Oxy-10 optodes (Presens, Germany). The oxygen consumption was converted to carbon units using a respiratory quotient of 1.0, which is slightly lower than the respiratory quotients reported from most lakes^36,37^, but close to estimates for rivers^36,38^ and river-sea interfaces^39,40^. We define short-term bioreactive DOC as the amount of DOC lost during the first 6 days of incubation (from day 0 to day 6), and medium-term bioreactive DOC as the carbon lost during the following 16 days (from day 7 to day 23). Both short- and medium-term bioreactive DOC pools were calculated from changes in dissolved oxygen concentration. In parallel, we incubated 1000 mL of 1.0 µm-filtered water (PALL A/E) in 1000-mL Duran glass bottles (dark, 20 °C) for 365 days to calculate DOC long-term bioreactivity, which we define as the DOC amount lost between day 24 and day 365. The DOC concentration was measured at Hatch Stable Isotope Laboratory, University of Ottawa, following standard procedures. The DOC long-term bioreactivity was calculated by subtracting the DOC concentration after 365 days from the DOC concentration at day 0 (collected on site) and by the DOC short- and medium-term bioreactive pools. Relative sizes of the different sub-pools of DOC were calculated as their proportions (%) of the initial DOC measured on day 0.

We also determined DOC optical properties such as CDOM (at 440 nm wavelength) and SUVA_254._ For the purpose, a volume of approximately 200 mL of sample was filtered with a 0.45-µm filter (30 mm PES filters, Thermo scientific, MA, USA) into 250-mL dark Thermo Scientific™ Nalgene™ amber bottle and stored in the fridge at approximately 1 °C until analyses, which were conducted following the method described in Selvam *et al*.^13^ (submitted).

Riverine gross primary production was calculated per unit of respiration (P: R ratio) according to Quay, *et al*.^41^, and described in detail by authors^42^. Accordingly, estimates on the balance between ecosystem productivity and respiration are based on the absolute mass ratio between ^18^O and ^16^O in the dissolved oxygen, water molecules, oxygen in air-water gas fluxes and oxygen in the air. Oxygen isotopes were determined at G.G. Hatch Stable Isotope Laboratory (Ottawa, ON, Canada) on a DELTA plus XL connected to a gas bench. In order to calculate ecosystem production, we assumed that the respiration measured *in vitro* at 20 °C degrees represented the *in situ* respiration, since the river water temperature was at a similar temperature (data not shown). The model represents production to respiration ratios for mixed water columns, which can be assumed for our rivers that are turbulent and not deep (ca 1.5–3 m at the sampling points). Moreover, we assumed that benthic respiration did not bias the production to respiration ratios. We then used the measured respiration rates multipled by the production : respiration ratio to derive phytoplankton primary production. Isotopic fractionation factors for respiration were applied following Bogard, *et al*.^43^. We obtained realistic production and respiration estimates for most systems, except for the upper Motala Ström, Ljusnan and Dalälven, which rendered unreasonable (negative) P: R ratio solutions, possibly due to analytical uncertainty in the δ^18^O determination of the dissolved oxygen^44^. To solve the three cases, we modified the input values by adding or subtracting maximum analytical errors, in different combinations. If more than one solution was found, the least extreme was chosen, i.e. the value closest to the mean values ratio between production and respiration in the other rivers.

We performed principal component analysis (PCA) on the proportions of land use coverage (Table 1) using XLSTAT 2017.5 (AddinSoft, Paris, France). Data were automatically centered and standardized with the PCA, and Varimax rotation was applied. We further conducted structural equation modeling using EQS. 6.3 (Multivariate Software, CA, USA) to test potential causal relationships between DOC short- and long-term bioreactivity and land use and WRT. This type of modeling tests the path significance between variables and also the overall significance of the modeled structure. Paths were considered significant when *p* < 0.05. The whole modeled structure was considered significant when *p* > 0.05, which indicates no significant differences between modeled and observed data. Model coefficients represent the slope of linear regressions between linked variables, however these coefficients can not be directly compared to common linear regressions, as their computation is different^45^. In order to attain the normal distribution, we applied a log (y + 1) transformation to principal component 1 data. Other non-normally distributed variables such as total phosphorus, WRT, STBR and LTBR were log transformed. Data were automatically centered and standardized during the SEM analyses, which makes the coefficients within a structure directly compared among each other. Higher coefficients represent stronger links between two variables.

## Supplementary information

Supplementary_Information

## Data Availability

Data is available from the corresponding author upon request.
