# Supplementary material for: Controls on Dissolved Organic Carbon Bioreactivity in River Systems
Source: Sci Rep. 2019 Oct 17;9:14897. doi: 10.1038/s41598-019-50552-y (PMC6797709; doi:10.1038/s41598-019-50552-y)
Supplement: Supplementary file 1 — Supplementary_Information [file 41598_2019_50552_MOESM1_ESM.docx]

**CONTROLS ON DISSOLVED ORGANIC CARBON BIOREACTIVTY IN RIVER SYSTEMS**

ANA R. A. SOARES^1*^, JEAN-FRANÇOIS LAPIERRE^2^, BALATHANDAYUTHABANI SELVAM^3^, GÖRAN LINDSTRÖM^4^, MARTIN BERGGREN^1^

1. DEPARTMENT OF PHYSICAL GEOGRAPHY AND ECOSYSTEM SCIENCE, LUND UNIVERSITY, SE-223 62 LUND, SWEDEN
2. UNIVERSITY OF MONTRÉAL, DEPARTMENT OF BIOLOGICAL SCIENCES, MONTREAL, PQ H3C 3J7, CANADA
3. DEPARTMENT OF THEMATIC STUDIES – TEMA ENVIRONMENTAL CHANGE, LINKÖPING UNIVERSITY, LINKÖPING, SWEDEN
4. SWEDISH METEOROLOGICAL AND HYDROLOGICAL INSTITUTE, NORRKÖPING, SE-601 76, SWEDEN

E-MAIL:ANARALVESSOARES@GMAIL.COM

**Supplementary Information**


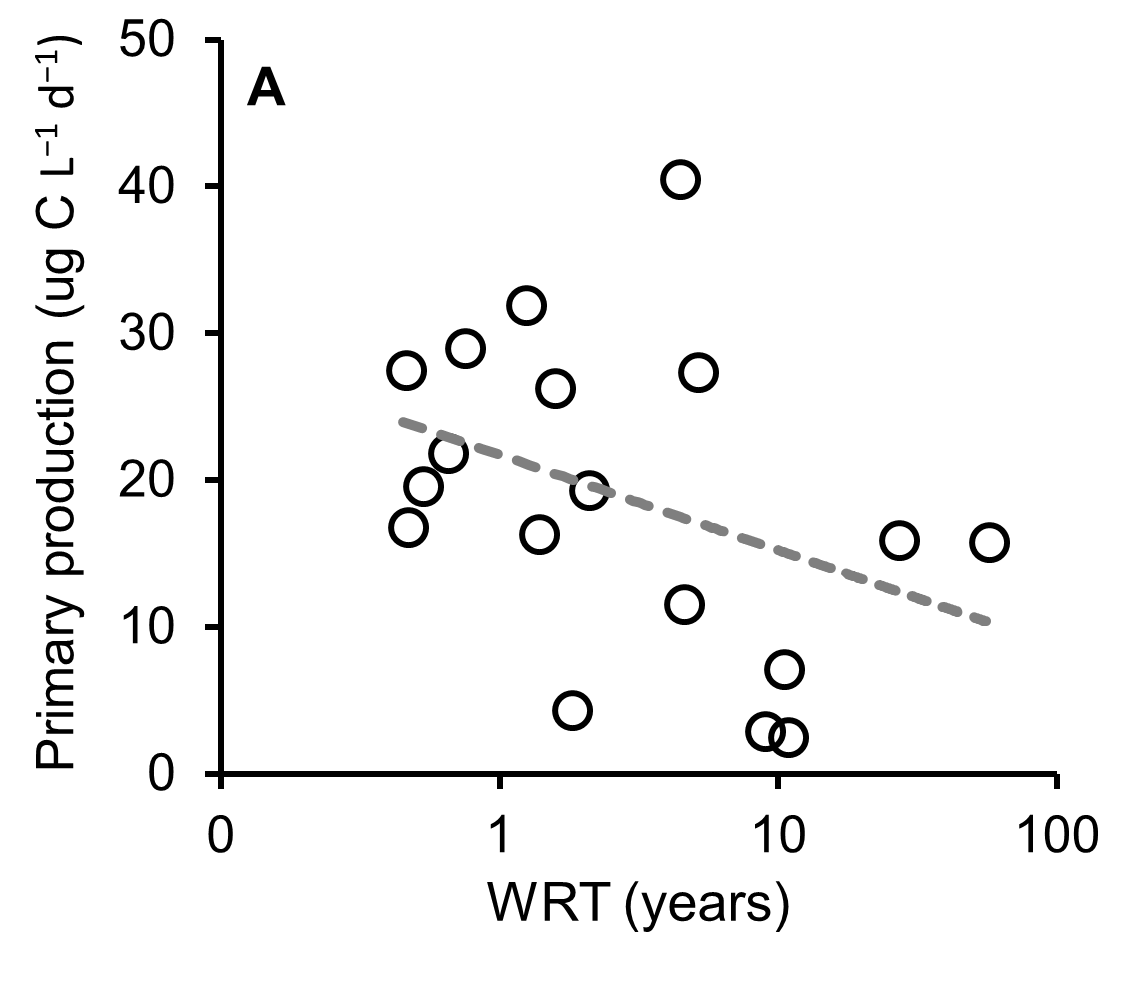


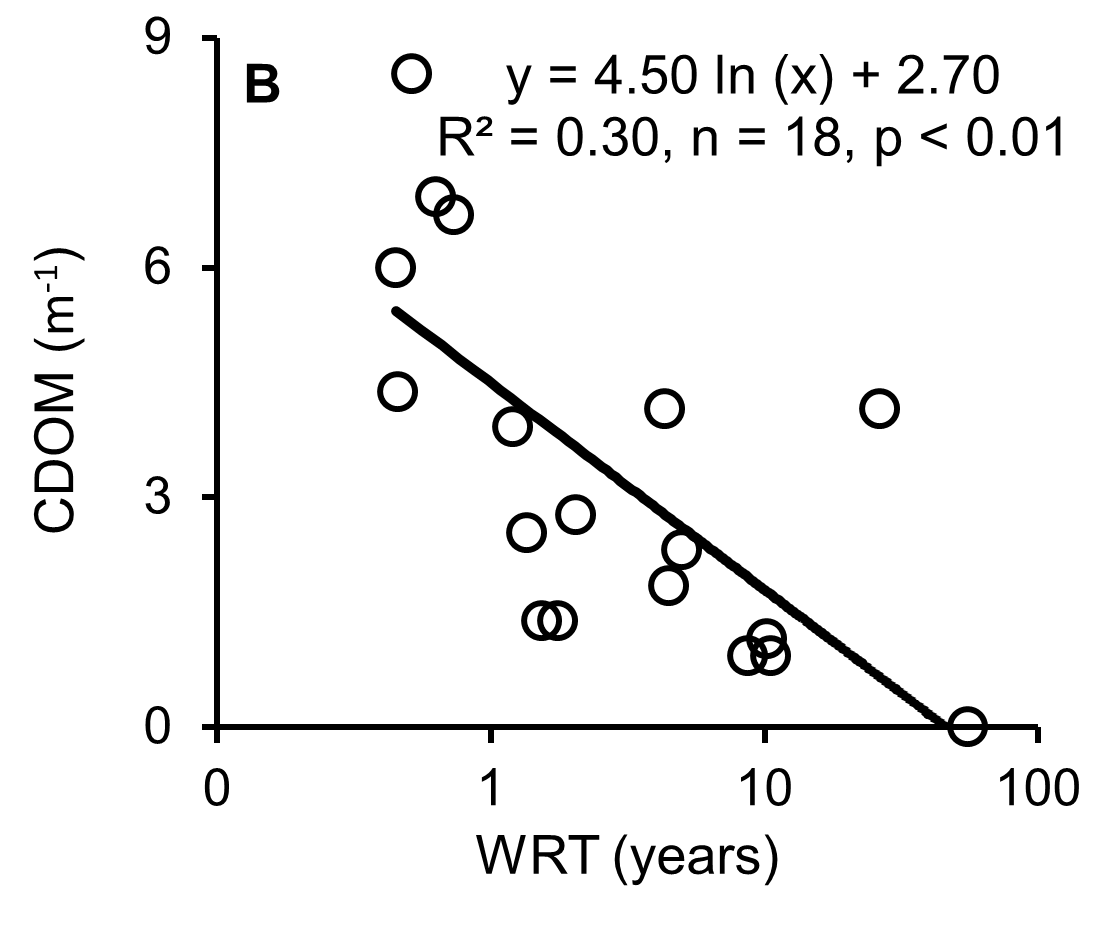


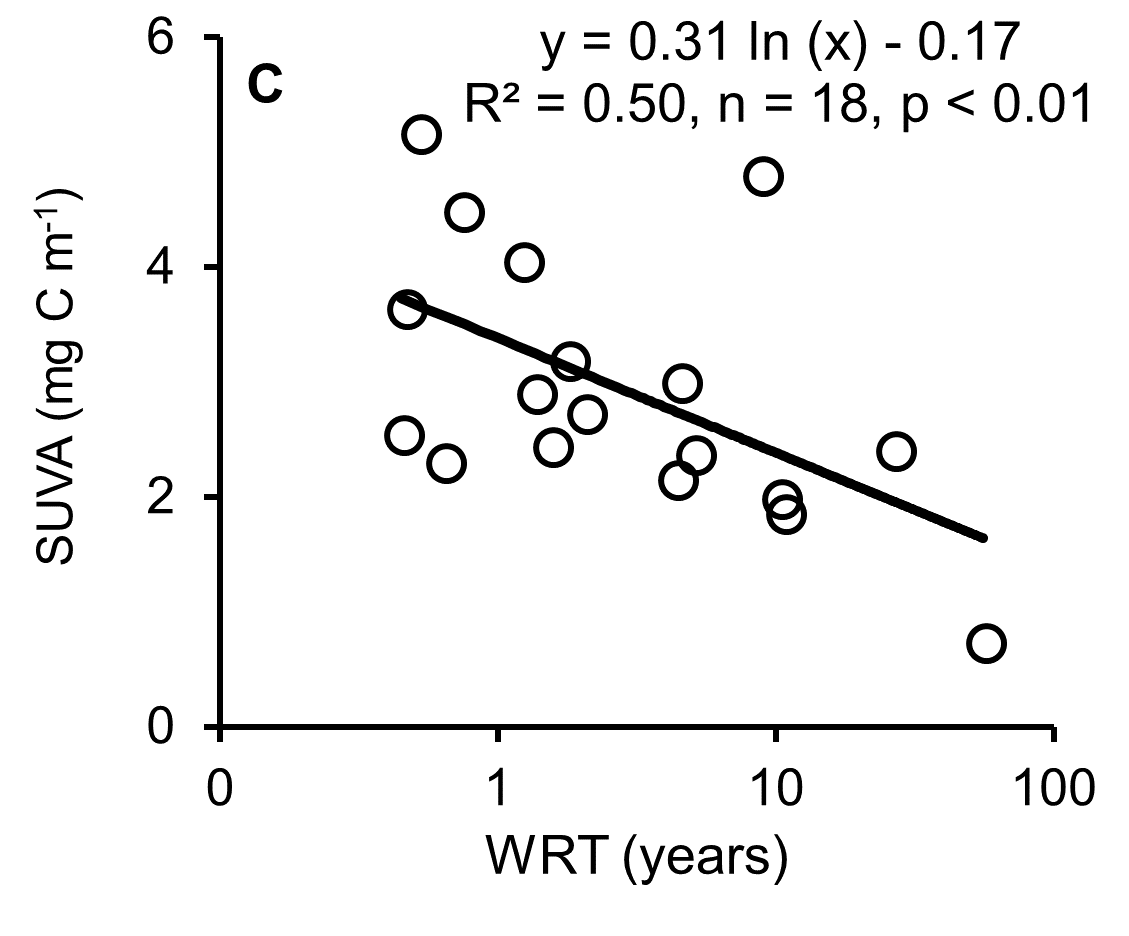


Supplementary Fig. 1. Primary production, specific ultraviolet absorbance at 254 nm (SUVA_254_) and coloured dissolved organic matter (CDOM) plotted against the logarithm of water residence time (WRT).


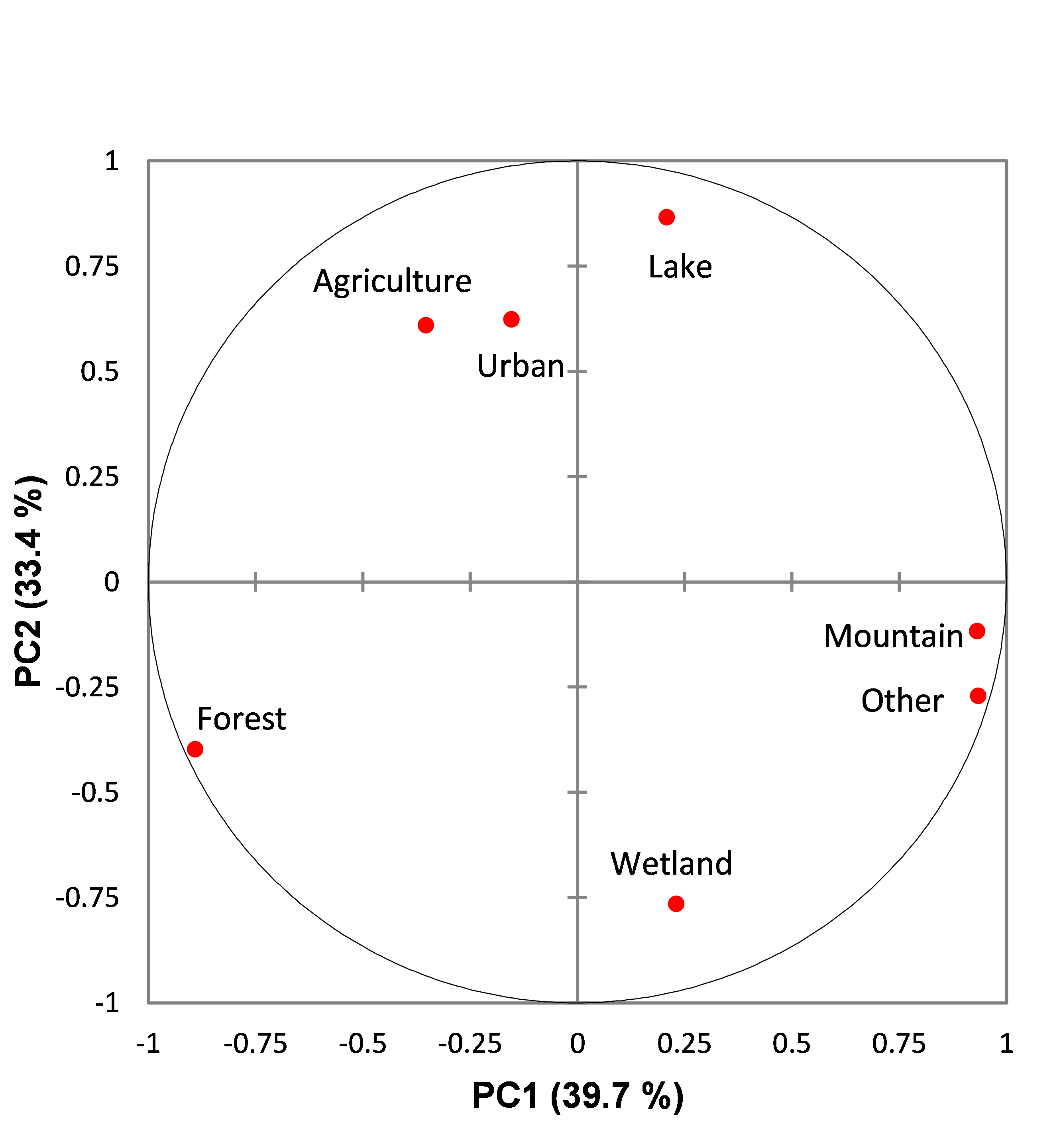


Supplementary Fig. 2. Principal components analysis on the percentage of land cover and land use categories (Table 1).


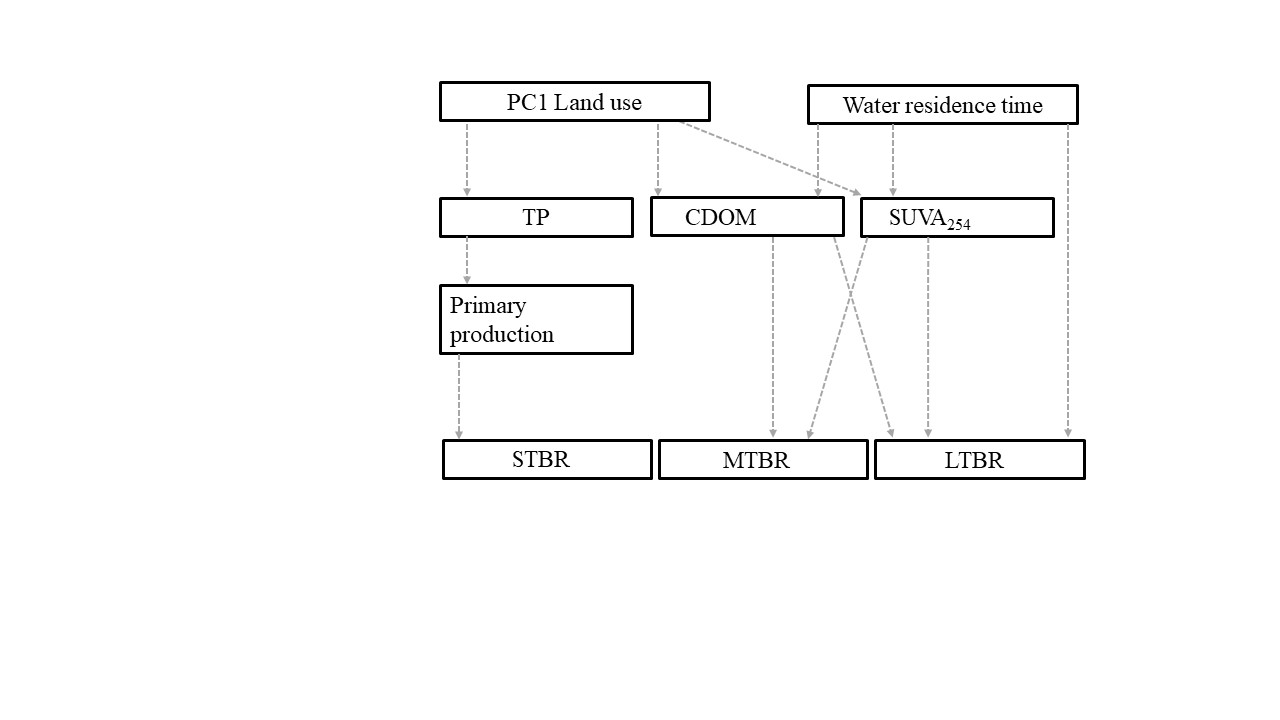


Supplementary Fig. 3. Structural equation model linking short- (STBR), medium- (MTBR) and long-term bioreactive (LTBR) pools to land use and water residence time (WRT). Dashed grey arrows denote significant pathways nonetheless the entire structure was non-significant (P < 0.05). χ^2^=64.21 ; P=0.00 ; df=27.

Supplementary Table 1. Primary production, specific ultraviolet absorbance at 254 nm (SUVA_254_) and coloured dissolved organic matter (CDOM) values for the 18 sampled sites.

| System  name | Primary  production  µg C L^-1^ d^-1^ | SUVA_254_  µg C m^-1^ | CDOM  m^-1^ | STBR  (mg L^-1^) | MTBR  (mg L^-1^) | LTBR  (mg L^-1^) | STBR  (%) | MTBR  (%) | LTBR  (%) |
| --- | --- | --- | --- | --- | --- | --- | --- | --- | --- |
| Torneträsk | 3.08 | 4.82 | 0.92 | 0.07 | 0.06 | 0.52 | 5 | 4 | 33 |
| Torne älv | 32.15 | 4.07 | 3.92 | 0.45 | 0.42 | 1.40 | 8 | 7 | 25 |
| Töre älv | 19.78 | 5.18 | 8.52 | 0.20 | 0.35 | 1.03 | 2 | 4 | 11 |
| Alterälven | 29.23 | 4.51 | 6.68 | 0.12 | 0.43 | 1.47 | 1 | 4 | 15 |
| Pite älv | 26.50 | 2.46 | 1.38 | 0.20 | 0.32 | 1.08 | 6 | 10 | 34 |
| Skellefte älv | 11.73 | 3.02 | 1.84 | 0.04 | 0.29 | 1.19 | 1 | 7 | 28 |
| Ume älv | 4.55 | 3.21 | 1.38 | 0.09 | 0.26 | 1.46 | 2 | 7 | 37 |
| Öre älv | 17.02 | 3.66 | 4.38 | 0.13 | 0.31 | 1.95 | 2 | 4 | 25 |
| Delångersån | 27.53 | 2.39 | 2.30 | 0.17 | 0.19 | 2.72 | 2 | 3 | 37 |
| Ljusnan | 16.55 | 2.92 | 2.53 | 0.16 | 0.26 | 2.56 | 2 | 3 | 34 |
| Dalälven | 19.52 | 2.74 | 2.76 | 0.12 | 0.26 | 2.92 | 2 | 4 | 39 |
| Nyköpingsån | 40.70 | 2.17 | 4.15 | 0.32 | 0.58 | 4.56 | 2 | 4 | 30 |
| Motala Ström | 16.11 | 2.42 | 4.15 | 0.15 | 0.45 | 4.31 | 2 | 5 | 44 |
| Vättern | 16.00 | 0.75 | 0.00 | 0.11 | 0.30 | 1.42 | 3 | 9 | 43 |
| Götä älv, Trollhättan | 2.71 | 1.88 | 0.92 | 0.08 | 0.14 | 1.51 | 2 | 3 | 30 |
| Götä älv, Alelyckan | 7.34 | 2.01 | 1.15 | 0.18 | 0.25 | 1.53 | 4 | 5 | 31 |
| Lyckebeån | 22.05 | 2.32 | 6.91 | 0.25 | 0.60 | 7.75 | 1 | 3 | 38 |
| Helge å | 27.67 | 2.57 | 5.99 | 0.27 | 0.58 | 5.82 | 2 | 3 | 33 |
